# Use of mouth rinse during pregnancy to improve birth and neonatal outcomes: a randomized controlled trial

**DOI:** 10.1186/s12884-015-0761-3

**Published:** 2015-11-25

**Authors:** Hong Jiang, Xu Xiong, Pierre Buekens, Yi Su, Xu Qian

**Affiliations:** School of Public Health; Global Health Institute, Fudan University, Mailbox 175, No. 138 Yixueyuan Road, Shanghai, 200032 China; Key Laboratory of Public Health Safety, Ministry of Education, Fudan University, Shanghai, China; School of Public Health and Tropical Medicine, Tulane University, 1440 Canal Street, Suite 2022, New Orleans, LA 70112 USA; Eye & ENT Hospital of Fudan University, Shanghai, China

**Keywords:** Birth outcomes, Mouth rinse, Pregnant women, Periodontal disease

## Abstract

**Background:**

Poor oral health, such as periodontal (gum) disease, has been found to be associated with an increased risk of adverse pregnancy outcomes including preterm birth, low birth weight, and neonatal and infant mortality, especially in low-and middle-income countries. However, there is little or no access to preventive dental care in most low-and middle-income countries. We propose to develop and test a “Mouth Rinse Intervention” among pregnant women to prevent the progression of periodontal disease during pregnancy and reduce adverse birth and neonatal outcomes in a rural county of China.

**Methods/Design:**

This is a randomized controlled clinical trial. A sample of 468 (234 in each arm of the study) women in early pregnancy with periodontal disease will be recruited for the study. Periodontal disease will be diagnosed through the methods of Periodontal Screening and Recording. All women diagnosed with periodontal disease will be randomly allocated into the intervention or control group. Women assigned in the intervention group will be provided with non-alcohol antimicrobial mouth rinse containing cetylpyridinium chloride throughout the pregnancy and oral health education. Women in the control group will receive a package of tooth brush and paste, plus oral hygiene education. Women will be followed-up to childbirth until the 42nd day postpartum. The main outcomes include mean birthweight (gram) and mean gestational age (week).

**Discussion:**

Compared with conventional mechanical ‘scaling and root planning’ periodontal treatment during pregnancy, our proposed mouth rinse intervention could be a simple, cost-effective, and sustainable solution to improve both mother’s oral health and neonate outcomes. If the mouth rinse is confirmed to be effective, it would demonstrate great potential for the application in other low- or middle-income countries to prevent adverse birth outcomes such as preterm birth and low birth weight and to reduce neonatal and infant mortality.

**Trial registration:**

This trial was registered with Chinese Clinical Trial Registry (ChiCTR): (#ChiCTR-TRC-13003768) on November 06, 2013.

## Background

Adverse birth outcomes, such as preterm birth and low birth weight, are the primary causes of infant morbidity and mortality in both developed and developing countries. Preterm birth complications lead to 35 % of world’s 3.1 million annual neonatal deaths [1]. A baby born with low birth weight (less than 2,500 g) had approximately 20 times risk to die than a baby with birth weight above 2,500 g [2]. Life course theory suggested negative birth outcomes including low birth weight and pre-term birth have lifelong effects, increasing the risk of chronic disease in adulthood [3]. As one of the developing countries, China is ranked as having the second highest (after India) number of neonatal and infant deaths in the world [4]. To tackle with and reduce the negative birth outcomes is an urgent task of achieving Millennium Development Goals and reducing susceptibility of adulthood chronic disease worldwide including China.

Systematic reviews have shown poor oral health, such as periodontal disease, is associated with an increased risk of adverse birth outcomes including preterm birth, low birth weight etc., especially in low- or middle-income countries [5–11]. But, several randomized controlled trials failed to show a significant reduction in preterm birth and low birth weight as a result of standard periodontal treatment in pregnant women with periodontitis [12–14].

As the standard therapy of periodontal disease, the scaling and root planning (SRP) during pregnancy itself possibly causes bacteremia thereby triggering a systemic inflammatory response, which may lead to adverse pregnancy and birth outcomes [15, 16]. Due to the safety reasons during pregnancy, the frequency of periodontal treatment are often restricted, with only one or two therapy courses, which might not sufficient to prevent the progression of periodontal disease [15, 17]. Therefore, it might be the SRP treatment itself that causes a failure of treating periodontal inflammations during pregnancy as well as preventing adverse birth outcomes.

In many poor and low-income areas, there is the universal shortage of qualified dental human resources [18–20]. The conventional SRP periodontal treatment is often unavailable for most of grass-roots. A simple, affordable and practical oral health care to prevent the progression of periodontal disease is needed. A recent clinical trial conducted in the U.S. has demonstrated that the use of non-alcohol antimicrobial mouth rinse containing cetylpyridinium chloride (CPC) results in a significantly reduced incidence of preterm birth and low birth weight in pregnant women with high risk, and that its use is safe to pregnant women [21]. Antimicrobial mouth rinse containing CPC is an attractive approach for gestational oral health care as its inexpensive cost and the capability of reducing bacterial plaque, gingival inflammation and periodontal disease without additional periodontal intervention [21]. In addition, it is easy to be implemented without the requirement of the operation by dental professionals [21]. However, this mouth rinse intervention has not been tested to prevent adverse birth outcomes in low resource settings, like in rural regions of China. Therefore, we proposed to conduct a randomized controlled trial (RCT) to examine whether a “Mouth Rinse Intervention (MRI)” during pregnancy would reduce adverse birth outcomes in a rural area of China.

### Objectives and hypotheses

The objective of this study is to develop and test aMRI among pregnant women to prevent the progression of periodontal disease during pregnancy and reduce adverse birth and neonatal outcomes in a rural county of Jiangxi Province, China.

### We hypothesize that the MRI will

- increase birth weight and prolong gestational duration;
- reduce progression of periodontal disease during pregnancy;
- improve neonatal health; and
- be acceptable and applicable in resource limited rural areas.

The primary outcomes are mean birth weight (grams) and mean gestational age (weeks). The secondary outcomes are periodontal index determined by the total code of periodontal examination in each sextant of the mouth, compliance of the use of antimicrobial mouth rinse, and rate of admission to Neonatal Intensive Unit (NICU).

## Methods/design

### Overall study design

**Fig. 1:**
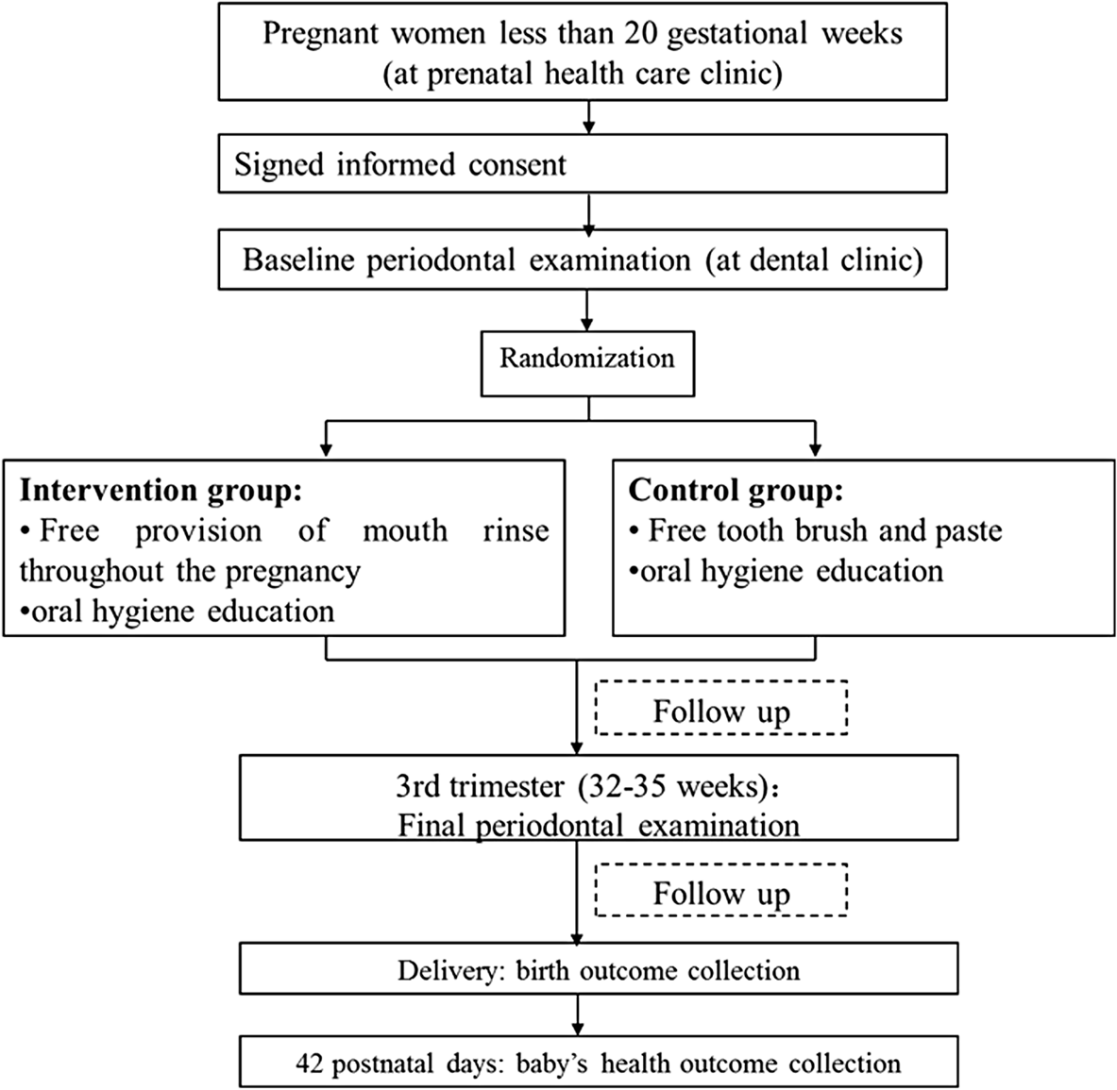
Research flow chart

This is a randomized controlled trial. Pregnant women less than 20 gestational weeks are eligible to participate in a quick periodontal disease screening. Women who meet the criteria and are diagnosed with periodontal disease will then be randomized into two groups: an intervention or MRI group, versus a control group. All participants will be followed throughout their pregnancy and their babies’ 42 postnatal days (see Fig. 1).

Ethical approval to conduct this trial has been granted by the Institutional Review Board of School of Public Health, Fudan University, Shanghai, China (IRB# 2013-09-0462); and also by the Institutional Review Board of Tulane University, New Orleans, Louisiana, USA (IRB# 13–469160). The research is registered with Chinese Clinical Trial Registry (#ChiCTR-TRC-13003768).

### Participants and recruitment

This RCT will be conducted at the Maternal and Child Health Care Hospital, in Leping, Jiangxi Province, China. Leping County locates in the southeast China, with a population of 900,000. The hospital is the only maternal and child health care hospital in the county, with a total of 4,000 deliveries annually (covering more than 40 % of deliveries in the county). There are both prenatal health care and dental clinic in the hospital.

Recruitment will be conducted among pregnant women attending prenatal health care at the hospital. All women attending prenatal health clinics, with less than 20 gestational weeks, will be approached and invited to participate in the study by research nurses. If pregnant women agree to participate in the study, they will be assessed for the eligibility. Eligible women will be provided with a free dental examination for screening periodontal disease. Women who meet the criteria of periodontal disease will be randomized into the intervention and control groups. Written informed consent will be obtained from each participant before the randomized group allocation is revealed.

### Inclusion criteria

- Pregnant women with gestational age less than 20 weeks
- At least 18 years old;
- Planning to deliver at the recruiting hospital

Additional inclusion criteria for randomization:

- Diagnosed as having periodontal disease after a dental examination
- At least 20 teeth
- Without moderate or severe dental caries
- Without systemic disease, including severe cardiovascular disease, diabetes, hyperthyroidism, and/or other systemic diseases
- Without reproductive disease such as infertility, and sexually transmitted diseases including syphilis, gonorrhoeae, trichomonas, mycotic vaginitis
- Without immunodeficiency diseases
- Willing to be compliant to follow up until baby’s 42 postnatal days

### Exclusion criteria

- Less than 18 years old
- Fewer than 20 teeth
- Contraindication to probing in a dental examination, such as heart disorders
- Unwilling or unable to sign the informed consent form
- Receiving periodontal treatment within the past six months
- With a disease mentioned in the inclusion criteria.

### Enrollment and randomization procedures

Random allocation to either the intervention or control group is decided by a computer generated random number. The group allocation will not be concealed until pregnant women have completed the dental examination, identified with periodontal disease and signed the informed consent. Research nurse will unseal the opaque envelopes and inform the women about the group allocation. A research group member who has no direct contact with participants will be responsible for generating the random numbers and preparing the envelopes.

### Intervention group

Women in the intervention group will be provided with free mouthwash (alcohol-free antimicrobial mouth rinse containing 0.7 % CPC) throughout the whole pregnancy and oral health education. All the women will be provided with detailed instructions regarding how to use the mouth rinse: twice daily for 30 seconds with the supplied rinse (after regular tooth brushing), and will be asked to keep a diary. This rinse mouthwash has been used in a study among pregnant women and has proved to be safe [21]. Pregnant women will be provided with the rinse mouthwash from the prenatal health care clinic at each month when they have the prenatal check-up. At the third trimester between 32 and 35 gestational weeks women in the intervention group will be requested to have dental re-examination. If women have preterm delivery prior to 32 gestational weeks, the measurement within two days of childbirth will be used to reflect the periodontal status during late pregnancy.

### Control group

Women in the control group will not receive antimicrobial mouth rinse but will instead receive a package consisting of tooth brush and paste, plus oral hygiene education. In the third trimester, women in the control group will be also asked to have a periodontal re-examination.

### Data collection

#### Baseline data

Prior to the periodontal examination, a questionnaire will be administered to collect information such as demographics, socio-economic status, medical and obstetric history, and oral hygiene, stress, etc.

### Periodontal screening and recording (PSR)

We will use a rapid Periodontal Screening and Recording (PSR) [22] tool for screening and identifying pregnant women with periodontal disease. PSR, developed by the American Academy of Periodontology, was a simple screening method for periodontal disease. In this method, the mouth is divided into sextants, using a ball tipped probe with a color-coded area 3.5 to 5.5 from the tip. The dentist inserts the probe into the periodontal pocket, walks around the circumference of each tooth, and observes the position of the color-coded band in relation to the gingival margin. Only the highest code obtained is recorded, representative for the code of the sextant it belongs to. Measurements are recorded in a special box chart [22–24]. The definition of different code is shown as in Table 1. Participant with at least one code of any sextant equal or above three will be diagnosed with periodontal disease [22]. Problems such as furcation involvement, mobility, muco-gingival issues, and recession should also be recorded.

**Table 1:** Definition of the code for each sextant in the mouth [22]

Code	Definition
0	The colored area of the probe is completely visible, representing healthy gingival tissues without bleeding on probing
1	The colored area of the probe is completely visible, without calculus or detective margins, but with bleeding on probing
2	The colored area of the probe is completely visible, with supragingival or subgingival calculus and/or detective margins.
3	The colored area of the probe is partly visible.
4	The colored area of the probe is completely invisible, indicating a probing depth greater than 5.5 mm.

All periodontal measurements will be performed by one dentist who will be blinded to the group allocation of pregnant women to ensure study reliability. A dental nurse will record the codes of periodontal screening and other conditions detected during the dental examination.

Prior to the enrollment, the dentist will be invited to participate in a calibration study. Five volunteers will be recruited and will be examined by both the research dentist and an experienced periodontist. Inter- and intra-examiner variations in codes of periodontal screening will be identified between the dentist and the experienced periodontist. They will discuss the difference and adjust the measurement approach until a final agreement is reached.

### Records of use of mouth rinse

All pregnant women in the intervention group will be provided with dairy form to record their daily usage of mouth rinse. At the time of dispensing the mouth rinse each month, the research nurse will collect the diary of the last month from pregnant women. We will calculate the percentage of women who have completed the scheduled use of mouth rinse (twice a day) to assess women’s acceptability and compliance of the use of the supplied mouthwash.

### Follow-up of birth and neonatal outcomes

We will follow up with the recruited women until their childbirth to evaluate their acceptability and compliance to the use of the mouth rinse and to assess their periodontal health status again before the childbirth. Medical records of pregnant women will be reviewed by the research nurse to extract information on birth outcomes including birth weight and gestational age. Prior pregnancy and reproductive history and adverse events during pregnancy will be documented. At the child health care clinic of 42 postnatal days, a questionnaire survey will be carried out by the research nurse to obtain newborns’ health information from birthday to 42 days postpartum.

### Sample size

#### Sample size calculation for primary outcomes

We calculated that a consecutive sample of 422 (211 in each arm of the study) pregnant women with periodontal disease will be required for the study, at 0.05 significance level with 80 % power. The sample size calculation is based on detecting changes in primary outcome of birthweight. To detect a difference in mean birthweight of 100 g between the groups, a sample size of 211 for each group is needed. This was based on an estimation of SD as 380 g in the intervention group and SD as 350 g in the control group. Given the estimated 10 % of loss to following up during pregnancy, a total of 468 women in early pregnancy with periodontal disease will be needed. With the estimation of 75 % prevalence of periodontal disease (from another study in urban area) [17], 624 women in early pregnancy will be recruited for periodontal disease screening. A total of 234 pregnant women with periodontal disease will be needed, at 0.05 significance level with 80 % power, if the sample size calculation is based on changes in mean gestational week to detect a difference of 0.5 week between two groups, with SD 1.5 week and 1.2 week in each group respectively. We use the larger sample size as the final amount for recruiting.

#### Calculation of statistical power for secondary outcomes

The power calculations for secondary outcomes include mean periodontal disease measurements—total periodontal code and rate of admission to NICU.

### Periodontal code

Given the initial sample size of 211 in each group for detecting differences in periodontal code between the intervention and control groups, the proposed study will have 99.9 % statistical power to detect a difference in periodontal code of 3, with a SD as 6 and 5 in each group respectively, at 0.05 significance level.

### Rate of admission to NICU

Given the sample size of 211 in each group, the proposed study will have 60.1 % statistical power to detect an Odds Ratio of 0.5 between two groups, with estimated rate of admission to NICU 15 % for the control group, at 0.05 significance level.

### Data management

Considering the nature of the intervention, participants will not be blinded to their group allocation. The dentist who is going to perform the baseline and final periodontal examination will be blinded for the group allocation.

All materials containing individual information of participants will be stored in a locked cabinet and only research team members will have the access. The computer with research information and data will be password protected and only authorized research team members will be able to access.

### Data analysis plan

Descriptive statistics will be performed to examine for all outcomes and covariates. For continuous variables, such as periodontal code, birth weight (gram), gestational age (week) will be compared using t-tests, or non-parametric equivalents for non-normally distributed variables. For categorical variables such as the rate of periodontal disease and rate of admission to NICU, chi-squared tests will be used. All outcomes will be compared between the intervention and control groups. All analyses will be conducted with “intention to treat” analysis [25].

### Data safety monitoring plan

A Data Safety Monitoring Plan and an independent Data Safety and Monitoring Board (DSMB) have been developed and established to ensure the safety of research participants and the validity and integrity of data. The major responsibilities of the DSMB are to develop protocol stopping guidelines related to the safety of individuals and the overall trial.

## Discussion

We proposed to conduct a RCT to assess a MRI among pregnant women to prevent the progression of periodontal disease during pregnancy and to improve birth and neonatal outcomes. As perinatal oral health care usually not available in rural China, our proposed intervention will be the first study aiming to explore a convenient, affordable, acceptable oral health care package in rural China. If the intervention is effective, it will be more acceptable than the conventional and standard periodontal therapy by women, and have potential generalizability to other similar poor areas, where childbearing aged or pregnant women have very limited access to oral health care.

The conventional periodontal therapy mainly includes “SRP,” which involves mechanically cleaning the teeth above and below the gum line by removing the etiologic agents that cause inflammation. However, due to the high cost, the demand for expensive dental equipment, and the need for professional dentists in order to perform periodontal therapy, as well as concerns regarding the safety of the therapy, this conventional periodontal therapy may not be feasible to apply in low resource settings. In addition, we use a rapid PSR for the screening periodontal disease. Compared to the conventional full-mouth dental examination, the PSR takes only a few minutes to conduct for each patient, does not require the use of expensive dental equipment, and can be performed by non-oral health professionals after receiving appropriate training, which is particularly applicable to economically poor regions. The proposed non-alcohol, 0.07 % CPC antimicrobial mouth rinse is commercially available and cheap; and it has been previously shown to be safe and to decrease the severity of periodontal disease and the rate of preterm birth and low birth weight [21].

Due to limited the research funding and study period, the sample size of this study is relatively small, not allowing for the observation of the birth outcomes as proportions e.g. the rate of low birth weight and the rate of preterm birth. If there is evidence of effectiveness after this initial study, we will seek further funding to expand the trial, with birth outcomes in proportions as primary outcomes.

In summary, our proposed MRI could be a simple, cost-effective, and sustainable solution to improve both mother’s oral health and neonate outcomes. If the mouth rinse is confirmed to be effective, it would demonstrate great potential for the application in other low- or middle-income countries to prevent adverse pregnancy outcomes such as preterm birth and low birth weight and to reduce neonatal and infant mortality.

## Abbreviations

CPC: cetylpyridinium chloride
DSMB: data safety and monitoring board
MRI: mouth rinse intervention
NICU: neonatal intensive unit
PSR: periodontal screening and recording
RCT: randomized controlled trial
SRP: scaling and root planning
